# Validation of the Affiliate Stigma Scale for Asian Indian American Dementia Family Caregivers

**DOI:** 10.1002/alz70858_102968

**Published:** 2025-12-24

**Authors:** Anju Wadhawan, Olga F Jarrín Montaner, Rula Btoush, Peijia Zha

**Affiliations:** ^1^ Rutgers, The State University of New Jersey, Newark, NJ, USA; ^2^ Rutgers, The State University, North Brunswick Township, NJ, USA; ^3^ Rutgers, The State University, Newark, NJ, USA

## Abstract

**Background:**

The Affiliate Stigma Scale is a commonly used instrument for measuring stigma experienced by caregivers of persons with intellectual disabilities and mental illness. It is particularly relevant within the Asian Indian community, where dementia may sometimes be considered a punishment for past life sins of the patient or their loved ones. Building on prior work to culturally adapt the affiliate stigma scale for Asian Indian American dementia family caregivers, this study aims to assess the reliability and validity of the culturally adapted scale.

**Method:**

Psychometric properties of the culturally adapted 22‐item Affiliate Stigma Scale were evaluated in a convenience sample of 222 Asian Indian dementia family caregivers living in the United States. Participants were recruited via personal and professional networks using email, social media, and flyers posted in adult senior centers, religious centers, ethnic grocery stores, community clinics, and cultural events. Data were collected using digital and paper surveys. Analyses included descriptive analysis of sample characteristics, factor analysis, reliability, and construct validity of the culturally adapted instrument.

**Result:**

The study sample consisted of participants who, for the most part, had not obtained a formal dementia diagnosis for their family member (55%). The majority had a college degree (80%), were employed full‐time (82%), and were under the age of 55 (73%). Half of the participants were male (53%), and half (50%) had lived in the U.S. for over 20 years. Participants traced their origins to 22 states in India, including Punjab (30%), Delhi (14%), Gujarat (29%), and Maharashtra (9%). The culturally adapted instrument had excellent reliability (Cronbach's alpha = 0.96). The culturally adapted instrument had excellent reliability (Cronbach's alpha = 0.96). After the initial exploratory factor analysis, confirmatory factor analysis revealed strong to moderate item relationships with the stigma construct (factor loading range 0.573 to 0.832) and supported a one‐factor solution.

**Conclusion:**

The adapted scale demonstrates strong reliability and validity, meeting standard psychometric criteria to provide meaningful and accurate results. The culturally adapted instrument is important for future research and clinical assessment of affiliate stigma in Asian Indian American dementia family caregivers.

